# The Effect of Inner Friction on Concrete Fracture Behavior under Biaxial Compression: A 3D Mesostructure Study

**DOI:** 10.3390/ma12233880

**Published:** 2019-11-24

**Authors:** Yi-Qun Huang, Shao-Wei Hu, Yue-Yang Sun

**Affiliations:** 1 College of Mechanics and Materials, Hohai University, Nanjing 210000, China; yiqunhuang@hhu.edu.cn; 2 School of Civil Engineering, Chongqing University, Chongqing 400044, China; 3 College of Water Conservancy and Hydropower Engineering, Hohai University, Nanjing 210000, China; syyang0118@126.com

**Keywords:** concrete, mesostructure, cohesive element, inner friction, biaxial compression, fracture

## Abstract

The mechanical behavior of concrete under biaxial loading condition (especially biaxial compression) is one of the most important indexes to evaluate the quality of concrete. To study the mechanical behavior of concrete under biaxial compression at mesoscale, we adopted our recently developed 3D numerical model based on Voronoi tessellation and cohesive elements. A constitutive model considering the friction effect is used in the model to characterize the fracture behavior of all potential fracture surfaces inside the concrete. A series of numerical experiments with different biaxial compression stress ratios were carried out. It was found that with the increase of the biaxial compression ratio, the proportion of energy increment caused by friction stress increases. The effect of inner friction coefficient on the biaxial relative strength was also investigated, and this kind of study is hard to be carried out through laboratory experiments. The results show that the inner friction coefficient has a great influence on the biaxial relative strength of concrete, and there is a positive correlation between these two parameters. Based on the above rules, a conservative biaxial relative compression strength envelope is obtained by setting the inner friction coefficient as zero.

## 1. Introduction

In the design of concrete structures, the biaxial strength and fracture behavior are very important. To reveal the mechanism of the concrete biaxial fracture, researchers have carried out a series of experiments [1,2,3,4,5,6,7], including biaxial compression experiments, biaxial tension experiments, and biaxial mixed tension compression experiments. In those studies, the researchers adopted the relative strength to reflect the strengthening effect (or weakening effect) of the complex loading condition. Based on the relative strength, the biaxial relative strength envelope was presented to evaluate the comprehensive bearing capacity of concrete. Some corresponding theories have also been proposed to calculate the strength and stress–strain relation [3,8,9,10]. Although the experimental and theory studies have been relatively established, the mechanical behavior of concrete under biaxial loading is less studied at mesoscale. Concrete is a composite material formed by aggregates, mortar, interface transition zone (ITZ), and some other contents. These meso contents can affect the macro mechanical behavior of concrete. Therefore, it is meaningful to study the mechanical behavior of concrete under biaxial loading conditions at mesoscale.

To study the mechanical behavior of concrete at mesoscale, we should establish an effective numerical model firstly. In recent years, many researchers have studied the meso behavior of concrete through different numerical models. The first issue to build up the numerical model is the generation of the aggregates. Two typical aggregate-generating methods have been proposed. The first method is to generate aggregates by randomly throwing the geometries [11,12,13,14], and the other method is to regenerate the original structure based on the tomography of concrete [15,16,17]. The second issue is to calculate the built up model, and many methods have been adopted, such as traditional finite element method [18,19], the lattice model [20,21,22], the homogeneous model (such as meso-element equivalent method) [23,24,25], the extended finite element method [26,27], and the scaled boundary finite element method [28,29,30]. The discrete element methods are also considered, such as the particle flow method [31,32,33] and the rigid-body-spring method [34,35].

Besides the method mentioned above, a modified finite element method with cohesive elements is beginning to be used by more and more researchers [36,37,38,39,40,41,42,43,44]. The cohesive element is an interface transition element between solid elements. Only the normal stress and tangential stresses can be transmitted in the cohesive element. The geometry thickness of the element is also independent to the calculation one, which means the geometry thickness can be set to any positive value, including zero. For this reason, more researchers have chosen this element to characterize the ITZ and potential fracture surfaces. The existing researches about the cohesive element mainly focus on the tensile fracture of concrete. However, the compression fracture behavior of concrete is much more complex. Particularly, the inner friction exists inside the concrete during the compression fracture process. The inner friction is a friction effect, which exists in the closed fracture surfaces inside the concrete. When the fracture surfaces of concrete are closed and subjected to compressive stress, the friction stress occurs once the fracture surfaces show the tendency of relative slip. Thus, it is necessary to consider the inner friction when studying the compression fracture behavior of concrete.

Based on our previous research works about the numerical model with cohesive element [43,44], in this paper, we adopt the Voronoi tessellation and zero thickness cohesive element to generate concrete mesostructure, and a modified constitutive model considering the inner friction effect is also adopted. On the basis of this numerical method, we investigate the fracture behavior of concrete under biaxial compression in terms of the mechanical behavior, fracture pattern, and energy evolution. Finally, the effect of inner friction coefficient on the biaxial relative strength is studied.

## 2. Numerical Model

### 2.1. 3D Mesostructure Based on the Voronoi Tessellation

**Figure 1:**
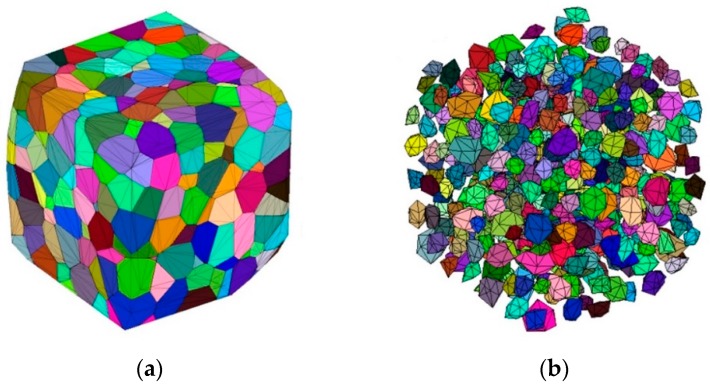
The typical Voronoi polyhedrons (**a**) and the generated aggregates (**b**) [44].

Due to the good shape of generated polyhedrons and the stability of calculation, Voronoi tessellation [45,46] is an effective method to generate the aggregates in 2/3D condition, and it has been applied in some numerical studies [14,38]. The Voronoi tessellation can generate seamlessly connected polyhedron cells based on the predefined seeds, and normally one seed can only generates one polyhedron, which provides a certainty for aggregate generation. In this paper, we adopt the aggregate-generating method which has been presented in our previous work [44]. The typical Voronoi polyhedrons and the generated aggregates are shown in Figure 1.

### 2.2. FE Discretization and Insertion of Cohesive Elements

Based on the generated aggregates, we can easily obtain the aggregate-mortar two-phase mesostructure. However, this two-phase structure is too simple to characterize the complex fracture behavior of concrete. Thus, a more effective numerical model should be established.

Cohesive element, as a special element type in the FE method, was developed to characterize the interface behavior of the traditional solid elements. This element can only transfer normal and shear stresses, and this characteristic is very suitable to characterize the mechanical behavior of fracture surfaces. The calculation thickness of cohesive element is also independent to its geometry thickness, and the geometry thickness can be set to any positive value, including zero, which means this element is very suitable to represent the components with extremely small thickness in concrete, such as ITZ. According to these characteristics, we chose zero thickness cohesive element to characterize the ITZ and potential fracture surfaces of concrete.

**Figure 2:**
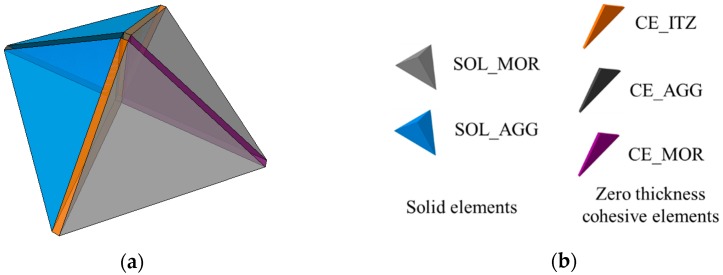
Different elements in concrete mesostructure: (**a**) the mesh with cohesive elements; (**b**) different types of elements in the mesh.

According to the algorism proposed in references [42,47,48], the cohesive elements are inserted into the mesh of two-phase mesostructure. The process of inserting cohesive elements can be divided into two steps: (1) split the continuous solid elements and renumber them (including element number and node number) and (2) insert the cohesive elements into the interfaces of those split solid elements. After inserting the cohesive elements, we can obtain a new mesostructure with five different elements. These five different elements (Figure 2) are: (1) solid elements for aggregates, namely SOL_AGG; (2) solid elements for mortar, namely SOL_MOR; (3) cohesive elements for ITZ, namely CE_ITZ; (4) cohesive elements for the potential fracture surfaces of mortar, namely CE_MOR; (5) cohesive elements for the potential fracture surfaces of aggregate, namely CE_AGG.

### 2.3. Constitutive Model Applied in Cohesive Elements

To characterize the mechanical behavior of ITZ and potential fracture surfaces (represented by cohesive elements), we adopted the modified constitutive model presented in our previous work with consideration of the friction effect between fracture surfaces [44], based on the mixed-mode damage model [49,50,51]. Here we give a brief introduction of this constitutive.

In the 3D case, one normal stress ($t_{n}$) and two orthogonal shear stresses ($t_{s}$, $t_{t}$) should be given during the calculation. In this paper, we define the calculation thickness of cohesive elements as 1. Thus, the constitutive model is controlled by stresses–displacements relation [52].

There are two phases that exist in the stresses–displacements relation: (1) elastic phase; (2) nonlinear phase. In the elastic phase, the relation between the stresses and displacements can be expressed as:

(1) $$t_{n}=\bar{k_{n}}\cdot \delta_{n}\;,\;t_{s}=\bar{k_{s}}\cdot \delta_{s}\;,\;t_{t}=\bar{k_{s}}\cdot \delta_{t}\;,$$

where $\bar{k_{n}}$ is the initial normal stiffness, $\bar{k_{s}}$ is the initial tangential stiffness, $\delta_{n}$ is the normal displacement, and $\delta_{s}$/$\delta_{t}$ is the orthogonal tangential displacement. The initial stiffness is an empirical parameter, and it should be determined in a reasonable range. According to the work done in [37,38,39], we set the value of the initial stiffness ($\bar{k_{n}}$, $\bar{k_{s}}$) as 10^6^ GPa in this paper.

The nonlinear phase initiates when the following condition is met:

(2) $${\left(\frac{\langle t_{n}\rangle }{t_{n0}}\right)}^{2}+{\left(\frac{t_{s}}{t_{s0}}\right)}^{2}+{\left(\frac{t_{t}}{t_{s0}}\right)}^{2}\geq 1\;,$$

where $t_{n0}$ is the normal strength, $t_{s0}$ is the shear strength, and 〈〉 is the Macaulay bracket.

**Figure 3:**
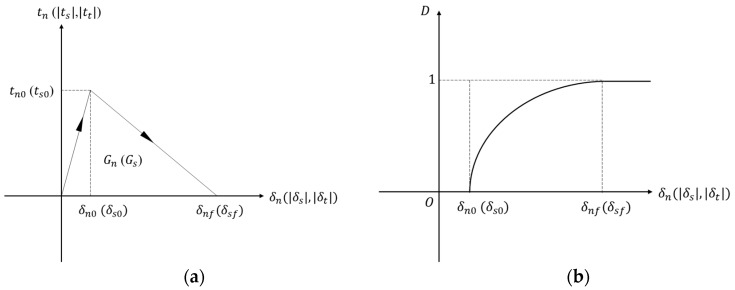
Bilinear model: (**a**) stress–displacement relation in single direction, (**b**) relation of damage factor and displacements.

As shown in Figure 3, the relation of stresses and displacements is characterized by bilinear model without consideration of friction. The area under the curve in Figure 3 means the fracture energy in normal (tangential) direction and can be calculated by:

(3) $$G_{n}=\frac{\delta_{nf}t_{n0}}{2},G_{s}=\frac{\delta_{sf}t_{s0}}{2}\;,$$

where $G_{n}$ is the normal fracture energy, $G_{s}$ is the tangential fracture energy, $\delta_{nf}$ is the normal failure displacement, and $\delta_{sf}$ is the tangential failure displacement.

The initial damage and failure surfaces are defined in the modified constitutive model to reflect the combined effect of normal and tangential damage. These two surfaces can be expressed as:

(4) $$\begin{array}{l} {\left(\frac{\langle \delta_{n}\rangle }{\delta_{n0}}\right)}^{2}+{\left(\frac{\delta_{s}}{\delta_{s0}}\right)}^{2}+{\left(\frac{\delta_{t}}{\delta_{s0}}\right)}^{2}=1\;\;(\mathrm{initial}\;\mathrm{damage}\;\mathrm{surface})\;,\\ {\left(\frac{\langle \delta_{n}\rangle }{\delta_{nf}}\right)}^{2}+{\left(\frac{\delta_{s}}{\delta_{sf}}\right)}^{2}+{\left(\frac{\delta_{t}}{\delta_{sf}}\right)}^{2}=1\;\;(\mathrm{failure}\;\mathrm{surface})\;, \end{array}$$

where $\delta_{n0}$ is the initial damage displacement in normal direction, and $\delta_{s0}$ is the initial damage displacement in tangential direction. The relation between $\delta_{n0}$ ($\delta_{s0}$) and $t_{n0}$ ($t_{s0}$) is shown in Figure 3.

**Figure 4:**
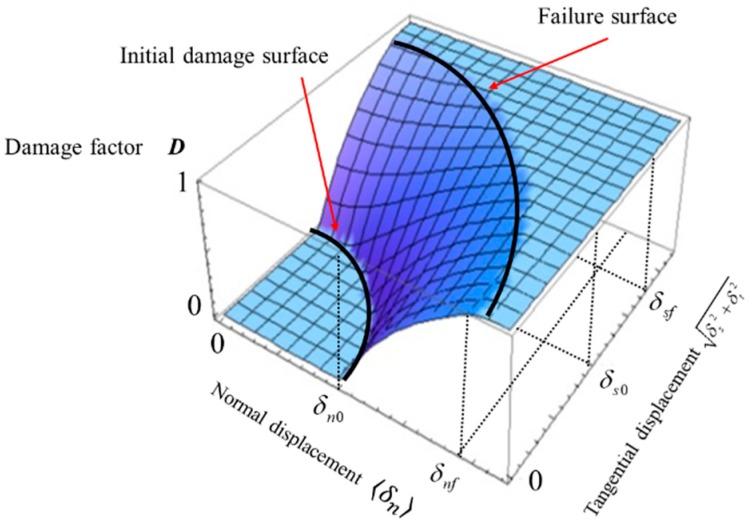
Relation between damage factor and displacements in the modified bilinear model.

According to the two damage surfaces mentioned above and the bilinear model, we can obtain the modified bilinear model with consideration of normal-tangential coupling effect. Figure 4 shows the relation of damage factor and displacements in the modified bilinear model.

**Figure 5:**
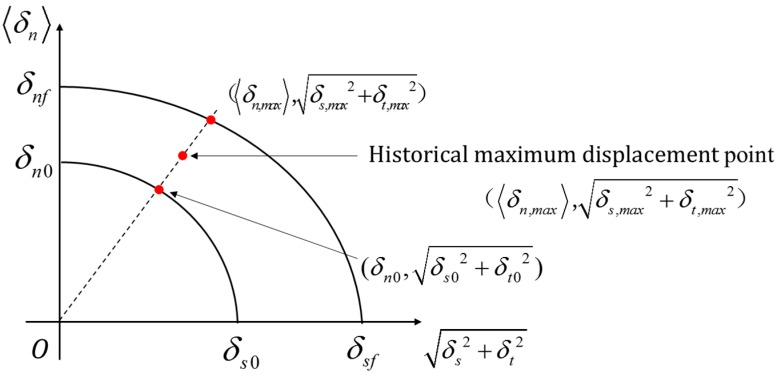
The relation between the intersection points on the damage surfaces and the historical maximum displacement point.

Based on the relation between damage factor and displacements, the damage factor *D* can be calculated through:

(5) $$D=\frac{(\delta_{max}-\delta_{0})\delta_{f}}{(\delta_{f}-\delta_{0})\delta_{max}}$$

where $\delta_{max}$ is the historical maximum loading displacement, $\delta_{0}$ is the relative initial damage displacement, and $\delta_{f}$ is the failure displacement. These parameters can be expressed as follows:

(6) $$\begin{array}{c} \delta_{max}=\sqrt{{\langle \delta_{n,max}\rangle }^{2}+\delta_{s,max}{}^{2}+\delta_{t,max}{}^{2}}\;,\\ \delta_{0}\;=\sqrt{\delta_{n,0}{}^{2}+\delta_{s,0}{}^{2}+\delta_{t,0}{}^{2}},\\ \delta_{f}=\sqrt{\delta_{n,f}{}^{2}+\delta_{s,f}{}^{2}+\delta_{t,f}{}^{2}}, \end{array}$$

where ($\delta_{n,max}$, $\delta_{s,max}$, $\delta_{t,max}$) is the normal and two orthogonal components of the historical maximum displacement point, ($\delta_{n,0}$, $\delta_{s,0}$, $\delta_{t,0}$) is the intersection point coordinates of the initial damage surface and the vector starting from the original point to historical maximum displacement point, and ($\delta_{n,f}$, $\delta_{s,f}$, $\delta_{t,f}$) is the intersection point coordinates of the failure surface and the vector mentioned above. The relation between these three points is shown in a plane formed by normal displacement and total tangential displacement (Figure 5), and we can easily calculate the coordinate values of two intersection points according to their geometry relationship (the specific expressions of $\delta_{n,0}$, $\delta_{s,0}$, $\delta_{t,0}$ and $\delta_{n,f}$, $\delta_{s,f}$, $\delta_{t,f}$ have been listed in our previous work [44]). 

Thus, without considering the friction effect, the relation between stresses and displacements can be expressed as:

(7) $$\begin{array}{l} t_{n}=\left\{ \begin{array}{l} (1-D)\bar{k_{n}}\delta_{n}\;\delta_{n}>0\;\\ \bar{k_{n}}\delta_{n}\;\delta_{n}\leq 0 \end{array}\right.\;,\\ \begin{array}{l} t_{s}=(1-D)\bar{k_{s}}\delta_{s}\;,\\ t_{t}=(1-D)\bar{k_{s}}\delta_{t} \end{array} \end{array}$$

**Figure 6:**
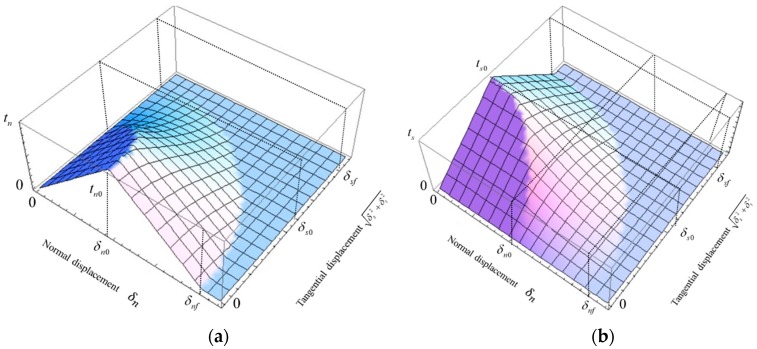
3D stress–displacements map without consideration of friction: (**a**) normal stress; (**b**) shear stress.

To better understand the relation between the stresses and displacements, a 3D stresses-displacements map is shown in Figure 6.

When the damaged area of cohesive element (fracture surfaces) is under compression, the friction occurs. The friction stress $T_{f}$ can be calculated through:

(8) $$T_{f}=\{\begin{array}{l} \tau_{max}\cdot \frac{\delta_{def}}{\left|\delta_{def}\right|}\;\tau_{max}\leq \bar{k_{s}}\cdot \left|\delta_{def}\right|\\ \bar{k_{s}}\cdot \delta_{def}\;\tau_{max}>\bar{k_{s}}\cdot \left|\delta_{def}\right|\; \end{array},$$

where $\tau_{max}$ is the maximum static friction stress, and $\delta_{def}$ is the tangential deformation displacement $\tau_{max}$ can be rewritten as:

(9) $$\tau_{max}=f\cdot \;\bar{k_{n}}\cdot \langle -\delta_{n}\rangle \;,$$

where f is the inner friction coefficient.

The tangential deformation displacement $\delta_{def}$ is calculated through:

(10) $$\delta_{def}=\sqrt{{\left(\delta_{s}-\delta_{s,slip}\right)}^{2}+(\delta_{t}-\delta_{t,slip}{)}^{2}}$$

where $\delta_{s,slip}$ and $\delta_{t,slip}$ are the orthogonal components of the relative slip displacement, and their initial values are zero when the simulation begins. When the calculation of the friction stress is done, the relative slip displacement should be updated if the relative slip occurs, and the updating formulations are:

(11) $$\begin{array}{l} \delta_{s,slip}^{new}=\delta_{s}-\frac{\tau_{max}}{\bar{k_{s}}}\mathrm{Cos}(\beta )\;(\tau_{\max}\leq \bar{k_{s}}\delta_{def})\;,\\ \delta_{t,slip}^{new}=\delta_{t}-\frac{\tau_{max}}{\bar{k_{s}}}\mathrm{Sin}(\beta )\;(\tau_{\max}\leq \bar{k_{s}}\delta_{def})\;, \end{array}$$

where $\delta_{s,slip}^{new}$ and $\delta_{t,slip}^{new}$ are the updated orthogonal slip displacement contents, and β is the direction of relative slip (also the direction of deformation). Cos(β) and Sin(β) can be calculated through:

(12) $$\begin{array}{l} \mathrm{Cos}(\beta )=\frac{\delta_{s}-\delta_{s,slip}^{old}}{\sqrt{{\left(\delta_{s}-\delta_{s,slip}^{old}\right)}^{2}+{\left(\delta_{t}-\delta_{t,slip}^{old}\right)}^{2}}}\;,\\ \mathrm{Sin}(\beta )=\frac{\delta_{t}-\delta_{t,slip}^{old}}{\sqrt{{\left(\delta_{s}-\delta_{s,slip}^{old}\right)}^{2}+{\left(\delta_{t}-\delta_{t,slip}^{old}\right)}^{2}}}\;, \end{array}$$

where $\delta_{s,slip}^{old}$ and $\delta_{t,slip}^{old}$ are the orthogonal slip displacement contents before being updated.

Thus, the orthogonal contents of friction stress can be rewritten as:

(13) $$T_{f,s}=T_{f}\cdot \mathrm{Cos}(\beta )\;,\;T_{f,t}=T_{f}\cdot \mathrm{Sin}(\beta )$$

Finally, the stresses of the cohesive element can be calculated through:

(14) $$\begin{array}{c} t_{n}=\left\{ \begin{array}{l} (1-D)\bar{k_{n}}\delta_{n}\;\delta_{n}>0\;\\ \bar{k_{n}}\delta_{n}\;\delta_{n}\leq 0 \end{array}\right.\;,\\ \begin{array}{l} t_{s}=(1-D)\bar{k_{s}}\delta_{s}+D\cdot T_{f,s}\;,\\ t_{t}=(1-D)\bar{k_{s}}\delta_{t}+D\cdot T_{f,t} \end{array} \end{array}$$

### 2.4. Extraction of Energies

Based on the constitutive model mentioned above, we can analyze the internal fracture situation by extracting the energies consumed inside the cohesive elements during the loading process. According to the stresses defined in the constitutive, three kinds of energy can be extracted, namely (1) the normal stress work caused by normal stress, (2) the shear stress work caused by shear stress, (3) the friction stress work caused by friction stress. These three kinds of energies can be calculated by:

(15) $$\begin{array}{l} E_{n}=\sum_{\mathrm{Cohesive}\;\mathrm{elements}}\iint_{A}(\int_{0}^{\delta_{n}}t_{n}d\delta )dA\;\;,\\ E_{s}=\sum_{\mathrm{Cohesive}\;\mathrm{elements}}\iint_{A}(2\int_{0}^{\delta_{s}}(t_{s}-D\cdot T_{f,s})d\delta )dA+\iint_{A}(2\int_{0}^{\delta_{t}}(t_{t}-D\cdot T_{f,t})d\delta )dA\;,\\ E_{f}=\sum_{\mathrm{Cohesive}\;\mathrm{elements}}\iint_{A}(2\int_{0}^{\delta_{s}}D\cdot T_{f,s}d\delta )dA+\iint_{A}(2\int_{0}^{\delta_{t}}D\cdot T_{f,t}d\delta )dA\;, \end{array}$$

where $E_{n}$, $E_{s}$, $E_{f}$ are the normal stress work, shear stress work, and friction stress work respectively, and A is the area of the cohesive element.

## 3. Numerical Samples and Results

### 3.1. Basic Simulation Information

#### 3.1.1. FE Input Data 

In this paper, we mainly study the fracture behavior of the concrete under biaxial compression. According to the previous works [1,2,3,4,5,6,7,8,9], the square plate is usually chosen as the shape of specimens under biaxial loading condition. For example, in the work done by Kupfer, H.B., et al. [1], Lee, S., et al. [5], and Karavelić, E., et al. [9], the size of the specimens are 200 mm × 200 mm × 50 mm, 200 mm × 200 mm × 60 mm, and 150 mm × 150 mm × 30 mm. For comparison purpose, we chose the concrete specimen with an appropriate size (150 mm × 150 mm × 30 mm) as the research object. The gradation of aggregate based on [53] is shown in Table 1 assuming the density as 2800 kg/m^3^. 

**Figure 7:**
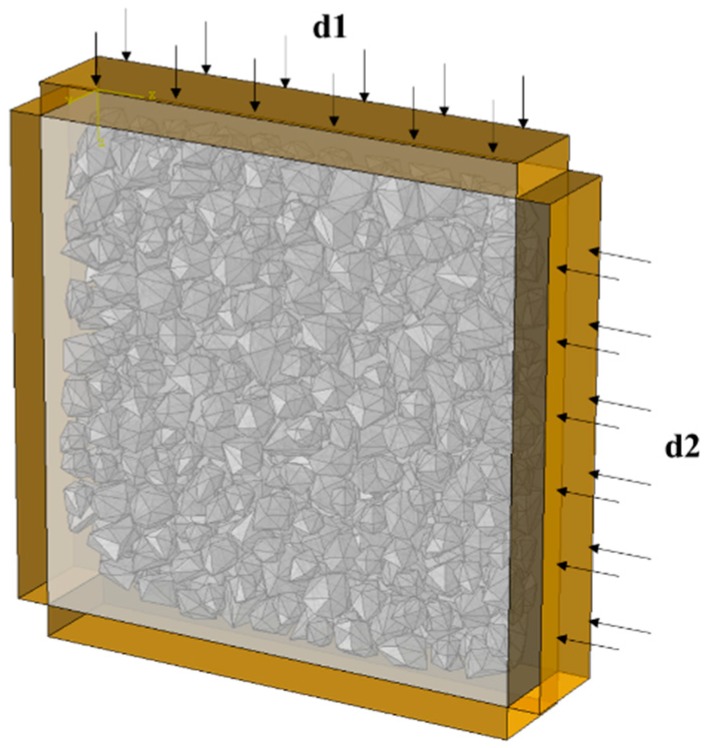
Loading scheme of concrete specimen under biaxial compression.

The loading scheme of the concrete specimen is shown in Figure 7. The concrete specimen is placed between two pairs of rigid plates, which are orthogonal. As shown in Figure 7, the rigid plates on the lower and left sides are fixed, and the rigid plates on the upper and right sides are applied by displacement loadings. The ratios of the two direction displacements in different test groups are shown in Table 2.

According to the previous works [1,2,3,4,5,6,7], the interaction between the rigid plates and concrete specimens should be set to a very low level to eliminate the effect of boundary constraints. Thus, we define the friction coefficient between the rigid plate and concrete as 0.001. 

**Figure 8:**
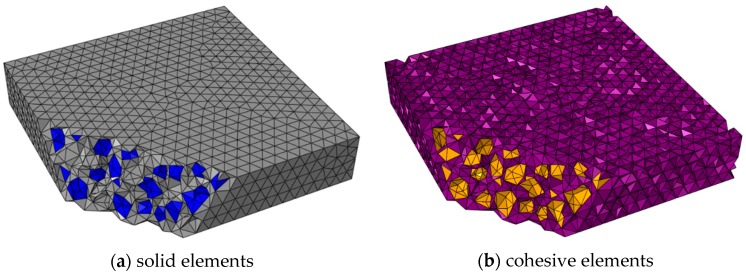
The mesh of a typical specimen: (**a**) solid elements with mortar in gray and aggregates in blue, (**b**) cohesive elements with mortar–mortar interfaces in purple and interface transition zone (ITZ) in orange.

The mesh of the specimen is shown in Figure 8, without the consideration of interfaces inside aggregates. The model is meshed appropriately through the repeat trial (average element size = 5 mm). 

According to the previous works about cohesive element model [41,42,43,44], repeated trial calculations, and the experimental results [53], the material parameters value applied in the solid elements are listed in Table 3.

**Figure 9:**
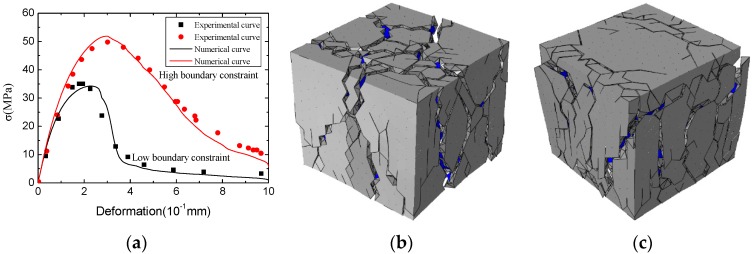
The mechanical and fracture behaviors of the concrete standard specimen [44]: (**a**) stress–strain curve, (**b**) the fracture pattern of concrete with low boundary constraint, (**c**) the fracture pattern of concrete with high boundary constraint (the deformation is magnified five times).

All numerical experiments were ended when the displacement d1 = 1.5 mm, corresponding to the macro strain ε = 0.01. Since the deformation of solid elements is small, and the strains of cohesive elements are equal to their displacements (the calculation thickness is set to 1), the small deformation formulation (nominal strain) is used. The numerical samples are solved in ABAQUS/EXPLICIT (Version 6.14-1, School of Civil Engineering, Chongqing University, Chongqing, China) solver with the user subroutine VUMAT [52]. By deleting the cohesive elements whose damage is equal to 1, the cracks of concrete can be represented. In our previous work [44], the numerical method adopted in this paper has proven that it can characterize the fracture and mechanical behavior of concrete appropriately, especially under uniaxial compression condition, as shown in Figure 9. Thus, we mainly discuss the behavior of concrete under biaxial compression condition in this paper.

#### 3.1.2. Mesh Convergence Analysis

**Figure 10:**
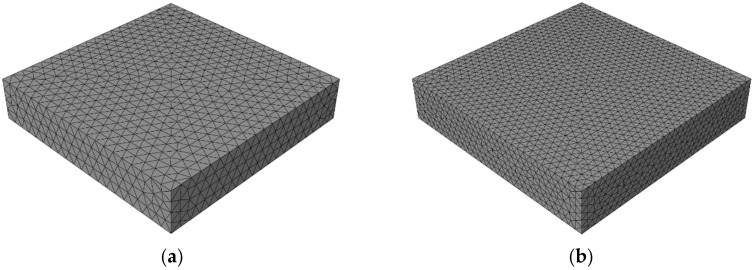
The mesh diagrams with different average element size: (**a**) 5 mm in Mesh I, (**b**) 3 mm in Mesh II.

In this paper, the potential fracture surfaces are represented by the pre-inserted cohesive elements, which means the cracks are forced to propagate following the faces of solid elements. For this reason, it is important to investigate the impact of mesh density on the simulation results. A test of mesh convergence was carried out, and models with different mesh densities are shown in Figure 10 (the average element size is 5 mm in Mesh I and 3 mm in Mesh II). In Mesh I, one typical model has about 130,000 nodes, 40,000 solid elements, and 60,000 cohesive elements. In Mesh II, one typical model has about 200,000 nodes, 70,000 solid elements, and 110,000 cohesive elements. These two models are subjected to a uniaxial compression loading (as shown in Figure 7, without the second direction displacement loading d2), and the displacement d1 = 1.5 mm. 

**Figure 11:**
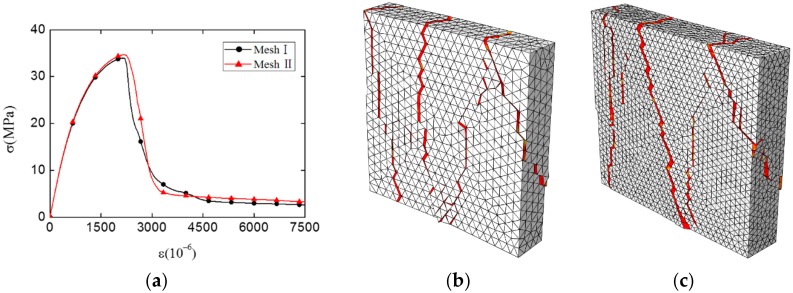
The results comparison between Mesh I and Mesh II: (**a**) stress–strain curve, (**b**) the fracture pattern of Mesh I, (**c**) the fracture pattern of Mesh II (the damaged cohesive elements are marked in red, and the deformation of specimen is magnified 1.5 times).

The simulation results of Mesh I and Mesh II are shown in Figure 11, including stress–strain curve and fracture pattern. Despite a few differences, the distribution of major cracks, the stress–strain curve, and the compression strength of two meshes are highly similar. Considering the balance of accuracy and efficiency, the element size of Mesh I was finally chosen for all meshes in the following simulations. 

#### 3.1.3. Quasi-Static Condition Examination

**Figure 12:**
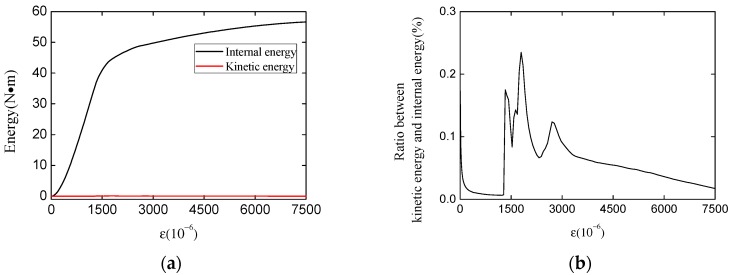
The evolution of internal energy and kinetic energy (**a**) and the ratio between kinetic energy and internal energy (**b**).

To ensure the quasi-static loading condition, the smooth step [52] is chosen, and the loading time is set to 1.5 s through the repeat trial. To examine whether the model meets the quasi-static loading condition, we extracted the kinetic energy and internal energy (the energy generated by deformation of the specimen) of a typical specimen under uniaxial compression condition. The evolution of kinetic energy and the internal energy during the loading process is shown in Figure 12. It can be seen that during the whole loading process, the ratio between kinetic energy and internal energy always keeps a very low value (under 0.3%). The results above show that the kinetic energy has little effect on the simulation results in this paper, which means that the numerical model in this paper meets the quasi-static loading condition. 

### 3.2. Typical Fracture Behavior of Concrete Specimens under Biaxial Compression

#### 3.2.1. Fracture Behavior 

**Figure 13:**
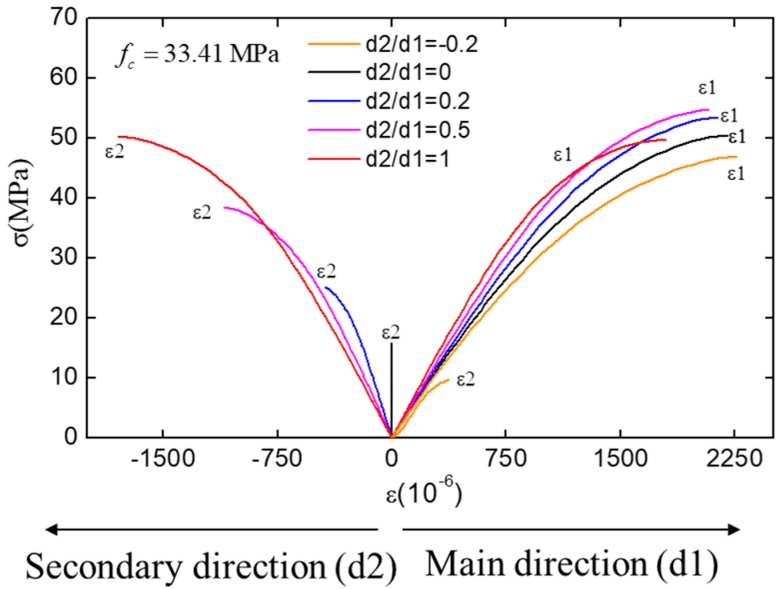
The stress–strain curves of concrete under biaxial compression.

The stress–strain curves of concrete under different biaxial compression conditions are shown in Figure 13. It can be seen that the relative strength of concrete under biaxial compression is much higher than the one under uniaxial compression in general, and the slope of curve in main direction (d1) increases with the increase of secondary-direction loading (d2). This phenomenon is caused by the restraint effect in secondary direction. Due to the restraint of the secondary direction, the concrete cannot deform freely in the plane formed by two loading direction. 

**Figure 14:**
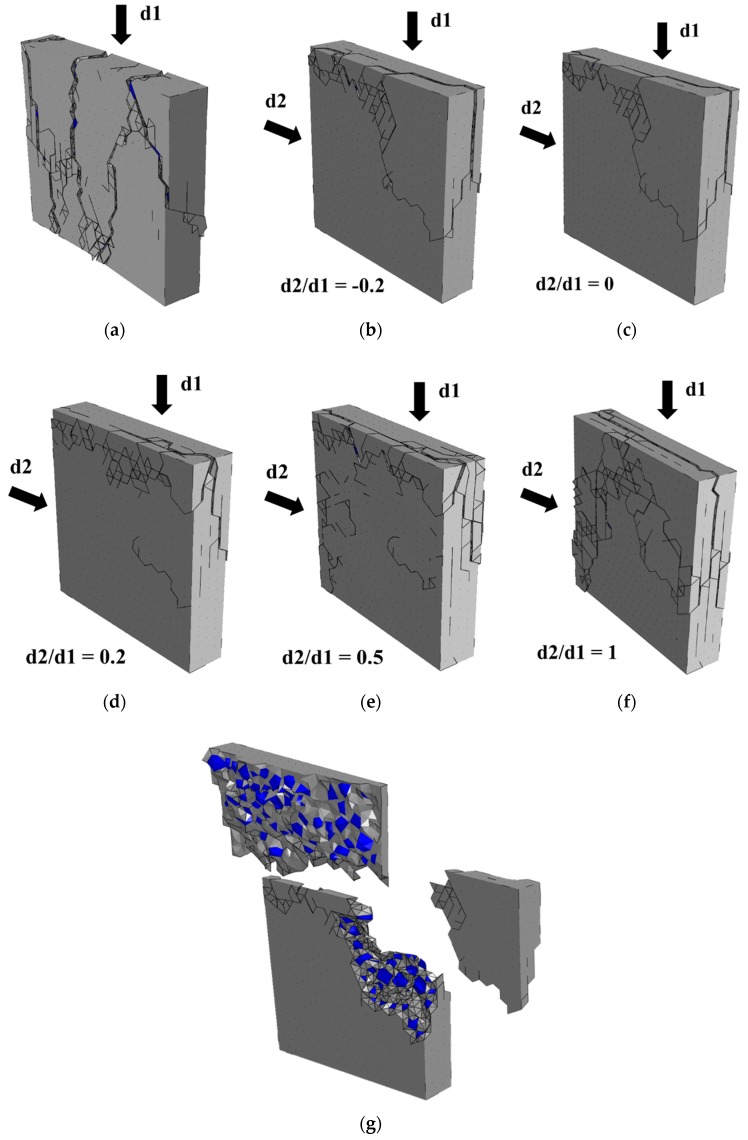
The fracture patterns of concrete under different loading conditions: (**a**) uniaxial compression, (**b**) biaxial compression d2/d1 = −0.2, (**c**) biaxial compression d2/d1 = 0, (**d**) biaxial compression d2/d1 = 0.2, (**e**) biaxial compression d2/d1 = 0.5, (**f**) biaxial compression d2/d1 = 1, (**g**) typical fragments of failure concrete specimen under biaxial compression (in (**a**–**f**), the deformation of specimen is magnified 1.5 times, and the fragments in (**g**) are obtained through the method introduced in reference [44]).

The fracture pattern (d1 = 1.5 mm) of concrete specimens under different loading conditions are shown in Figure 14. In the uniaxial compression condition, the concrete specimen shows an in-plane failure, and all cracks are basically parallel to the thickness direction. In the biaxial compression conditions, the concrete specimen shows an out-of-plane failure, and all the cracks are basically forming a slight angle to the plane formed by two loading directions (as shown in Figure 14g). This is because the deformation in two loading directions is restrained, and the cracks can only expand in the third direction. Moreover, with the increase of the biaxial stress ratio, more cracks occur in concrete.

#### 3.2.2. Energy Analysis

**Figure 15:**
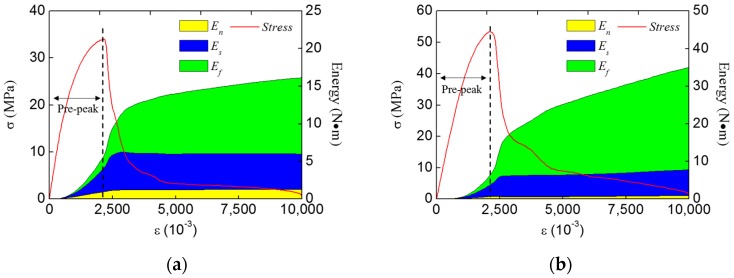
The typical energy evolution of concrete specimen during the fracture process under different conditions: (**a**) uniaxial compression condition, (**b**) biaxial compression condition (d2/d1 = 0.2)**.**

To investigate the fracture modes, the energy analyses were carried out. Figure 15 shows the typical energy evolution of concrete specimen during the fracture process under uniaxial/biaxial compression condition. It can be seen that the shear stress work and friction stress work dominate the whole fracture process in both loading conditions, and the normal stress work always keeps a low value. Compared to the one under uniaxial compression condition, the friction stress work accounts for a larger proportion under the biaxial compression condition, meanwhile, the normal stress work and shear stress work have a smaller proportion.

**Figure 16:**
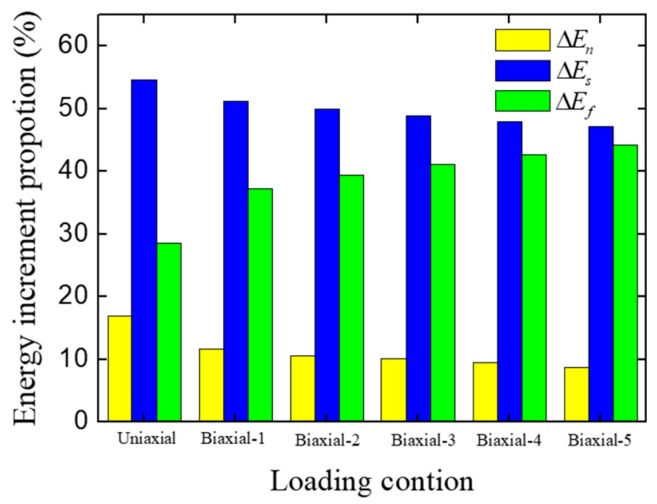
Energy increment proportions of concrete specimen under different loading conditions at the pre-peak stage.

To analyze the effect of the biaxial-loading ratio on the concrete fracture mode in terms of energy, we extracted the energy increments at the pre-peak stage, and these are listed in Table 4. The proportion of different kinds of energy increment are also shown in Figure 16. With the increase of biaxial-loading ratio, the proportion of friction stress work increases, meanwhile the normal stress work and shear stress work decrease. This is because of the increase of constraint on the loading surfaces, and the boundary constraint makes the concrete specimen separate more hardly. For this reason, more cracks are closed and subjected to the compression stress. When the cracks slip, more friction work is generated.

### 3.3. Effect of Inner Friction Coefficient

**Figure 17:**
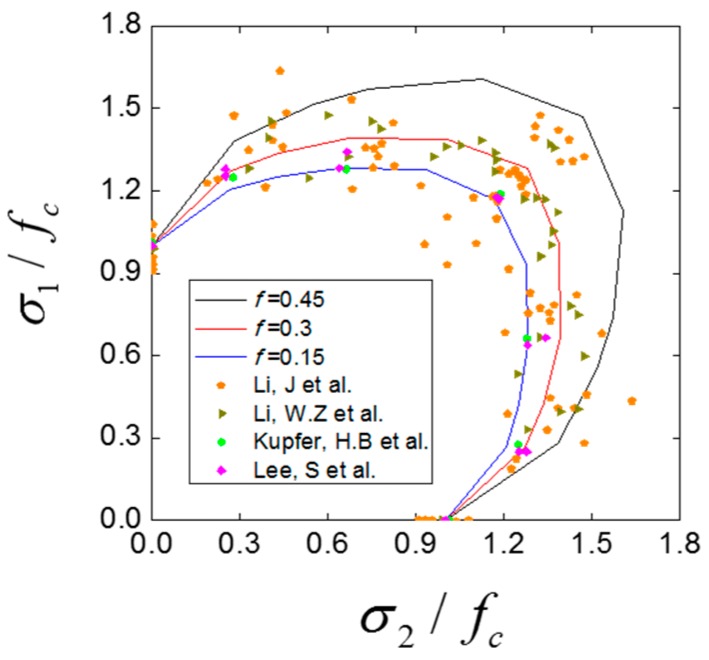
The biaxial relative strength envelopes with different inner friction coefficient together with the experimental results [1,3,5,8].

**Figure 18:**
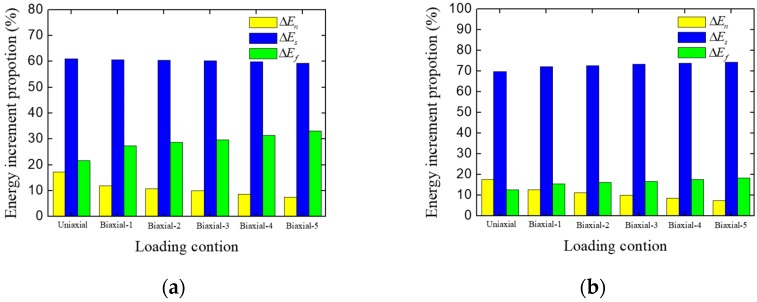
Energy increment proportions of concrete specimen with different inner friction coefficient: (**a**) *f* = 0.3; (**b**) *f* = 0.15.

Here we investigate the effect of inner friction coefficient on the biaxial strength envelope. Figure 17 shows the biaxial relative strength envelopes with different inner friction coefficient, together with the experimental data obtained by Kupfer, H.B., et al. [1], Li, W.Z., et al. [3], Lee, S., et al. [5], Li, J., et al. [8]. It can be seen that the numerical results show an agreement with the experimental one. The region contained in the envelope also decreases with the decrease of inner friction coefficient. This phenomenon shows that the inner friction coefficient has a great influence on the relative strength of concrete under biaxial compression condition. When the inner friction coefficient decreases, the fracture surfaces inside concrete can slip with less friction work consumption (as shown in Figure 18, the proportion of friction work decreases with the decrease of inner friction coefficient), which means concrete can be destroyed at a lower strength. 

**Figure 19:**
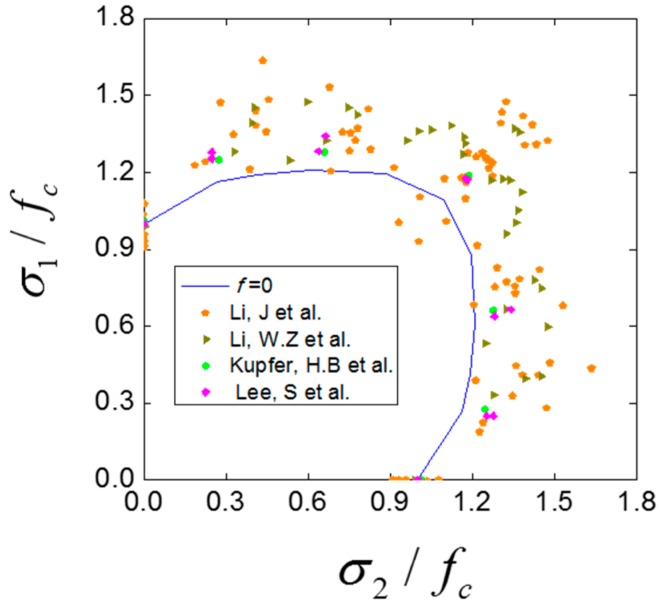
The biaxial relative strength envelopes with zero inner friction coefficient together with the experimental results [1,3,5,8].

Since the inner friction coefficient has a great effect on the biaxial relative strength envelope, we carried out a group numerical tests with zero inner friction coefficient to find out the biaxial compressive capacity of concrete without inner friction, and this kind of test is impossible to carry out in laboratory condition. As shown in Figure 19, the max relative strength remains at about 1.18–1.2, and the biaxial relative strength envelope is basically on the inner edge of those experimental data. Thus, we have obtained a relatively conservative biaxial relative compression strength envelope, and this envelope can be used as a reference in the design of concrete structures. 

## 4. Conclusions

In this paper, we build up a 3D meso model of concrete based on the Voronoi tessellation and cohesive element. A modified cohesive constitutive model considering the inner friction effect is applied in the model. Based on the proposed model, the biaxial compression behavior of concrete was investigated by analyzing the fracture pattern and energy evolution. The numerical results show an agreement with the experimental one. Several conclusions can be obtained.

(1) Inner friction (the friction effect which exists in the closed fracture surfaces inside the concrete) has a great effect on biaxial relative compression strength envelope. The relative strength increases with the increase of inner friction coefficient. 

(2) A conservative biaxial relative compression strength envelope is obtained through a group of numerical tests with zero inner friction coefficient. This biaxial relative strength envelope can be used as a reference in the design of concrete structures.

In this study, we mainly investigate the effect of inner friction on the concrete biaxial compression behavior. However, there are still many issues need to be studied on the basis of this research. For example, the fracture behavior of concrete under other complex loading conditions is still worth studying, and we can carry out a series of numerical tests to reveal its meso-mechanism. 

## Figures and Tables

**Table 1:** The gradation of aggregates [53].

Grain Size (mm)	Unit Content (kg/m^3^)	Volume Content (%)
4–8	540	19.29
2–4	363	12.91
1–2	272	9.71
0.5–1	272	9.71
0.25–0.5	234	8.35

**Table 2:** Design of numerical experiments.

Group	Ratio of Displacements of Two Direction d2/d1
Uniaxial	—
Biaxial-1	−0.2
Biaxial-2	0
Biaxial-3	0.2
Biaxial-4	0.5
Biaxial-5	1

Note: “—” means the parameter (d2/d1) is not required in the corresponding group.

**Table 3:** Material parameters applied in the concrete.

Element Type	$E\;(\bar{k_{n}}$, $\bar{k_{s}})\;(\mathrm{GPa})$	*v*	$t_{n0}\;(\mathrm{MPa})$	$t_{s0}\;(\mathrm{MPa})$	$G_{n0}\;(N\cdot m)$	$G_{s0}\;(N\cdot m)$	*f*
SOL_MOR	25	0.2	—	—	—	—	—
SOL_AGG	70	0.2	—	—	—	—	—
CE_MOR	10^6^	—	3	10.5	40	400	0.45
CE_ITZ	10^6^	—	1.5	5.25	20	200	0.45

Note: *E* for solid elements and $\bar{k_{n}}$, $\bar{k_{s}}$ for cohesive elements, and “—” means this parameter is not required in the corresponding element”.

**Table 4:** Energy increments and proportion of concrete specimens at the pre-peak stage.

Experiment Group	Energy Type	Energy Value (N∙m)	Energy Proportion (%)
Uniaxial	$\Delta E_{n}$	0.96	16.90
	$\Delta E_{s}$	3.10	54.58
	$\Delta E_{f}$	1.62	28.52
Biaxial-1	$\Delta E_{n}$	0.72	11.69
	$\Delta E_{s}$	3.15	51.41
	$\Delta E_{f}$	2.29	37.18
Biaxial-2	$\Delta E_{n}$	0.66	10.63
	$\Delta E_{s}$	3.10	49.92
	$\Delta E_{f}$	2.45	39.45
Biaxial-3	$\Delta E_{n}$	0.66	10.03
	$\Delta E_{s}$	3.21	48.78
	$\Delta E_{f}$	2.71	41.19
Biaxial-4	$\Delta E_{n}$	0.77	9.41
	$\Delta E_{s}$	3.92	47.92
	$\Delta E_{f}$	3.49	42.67
Biaxial-5	$\Delta E_{n}$	0.91	8.65
	$\Delta E_{s}$	4.96	47.15
	$\Delta E_{f}$	4.65	44.20
